# Diagnostic accuracy and association with lymph node metastasis of the systemic immune-inflammation index in thyroid cancer: a systematic review and meta-analysis

**DOI:** 10.3389/fimmu.2026.1852817

**Published:** 2026-06-26

**Authors:** Ruxandra Stefania Dragota, Michael Schenker, Ana-Maria Ciurea, Adina Turcu-Stiolica, Mihaela Popescu, Andreea Stanculescu, Bogdan Silviu Ungureanu, Tudorel Ciurea

**Affiliations:** 1 Doctoral School, University of Medicine and Pharmacy of Craiova, Craiova, Romania; 2 Oncology Department, Sf. Nectarie Oncology Center, Craiova, Romania; 3 Department of Oncology, University of Medicine and Pharmacy of Craiova, Craiova, Romania; 4 Biostatistics Department, University of Medicine and Pharmacy Craiova, Craiova, Romania; 5 Department of Health Economics and Outcomes Research, Faculty of Medicine, University of Medicine and Pharmacy “Iuliu Hatieganu”, Cluj-Napoca, Romania; 6 Department of Endocrinology, University of Medicine and Pharmacy of Craiova, Craiova, Romania; 7 Department of Anesthesiology and Intensive Care, Faculty of Medicine, University of Medicine and Pharmacy of Craiova, Craiova, Romania; 8 Gastroenterology Department, University of Medicine and Pharmacy of Craiova, Craiova, Romania

**Keywords:** diagnostic, LNM, SII, systemic immune-inflammation index, thyroid cancer

## Abstract

**Background:**

The Systemic Immune-Inflammation Index (SII), a composite biomarker integrating neutrophil, platelet, and lymphocyte counts, has been increasingly investigated in thyroid cancer. However, its diagnostic accuracy and association with lymph node metastasis (LNM) remain uncertain. This study aimed to systematically evaluate the diagnostic performance of SII for thyroid cancer and its association with LNM.

**Methods:**

A systematic literature search was conducted across PubMed, Scopus, and Web of Science. A diagnostic meta-analysis using the bivariate Reitsma model was performed to pool sensitivity, specificity, likelihood ratios, and the area under the summary receiver operating characteristic curve (AUC). The association between SII and LNM was assessed by pooling mean differences (MD) using an inverse-variance random-effects model. Heterogeneity was evaluated using I² statistics and Cochran’s Q test. This systematic review was registered in PROSPERO (PROSPERO 2025 CRD420251233710).

**Results:**

Ten studies were included. The diagnostic meta-analysis (6 studies; 2,209 participants) yielded a pooled sensitivity of 76.8% (95% CI: 63.8–86.1%), specificity of 71.2% (95% CI: 54.9–83.4%), and AUC of 0.805. The positive and negative likelihood ratios were 2.78 and 0.34, respectively. The LNM meta-analysis (5 studies; 2,073 participants) demonstrated significantly higher SII in patients with LNM (MD = 102.87; 95% CI: 58.86–146.89; p < 0.00001; I² = 56%). Leave-one-out sensitivity analyses confirmed the robustness of both findings. Sensitivity analysis excluding one outlier study yielded consistent results with reduced heterogeneity (MD = 105.72; I² = 32%).

**Conclusion:**

SII demonstrates moderate diagnostic accuracy for thyroid cancer and is significantly elevated in patients with lymph node metastasis, supporting its potential as an adjunctive biomarker for diagnostic triage and preoperative risk stratification. Given substantial between-study heterogeneity, threshold variability, and partial verification bias affecting specificity, the pooled diagnostic estimates should be interpreted as preliminary and require confirmation in prospective studies with standardized cut-offs and uniform histopathological reference standards. Further large-scale prospective studies are warranted.

**Systematic review registration:**

https://www.crd.york.ac.uk/prospero/, identifier CRD420251233710.

## 1 Introduction

Thyroid cancer is the most common malignancy of the endocrine system, with an estimated 821,214 new cases worldwide in 2022 (1). Its incidence has risen over the past decades, predominantly driven by an increase in papillary thyroid carcinoma (PTC), which represents over 85% of all thyroid cancer cases (2). Although the overall prognosis is favorable, with a five-year survival rate of approximately 98.5%, outcomes vary significantly according to histopathological subtype and disease stage at diagnosis (1).

In current clinical practice, ultrasound examination represents the primary imaging modality for thyroid nodule evaluation, with fine-needle aspiration biopsy (FNAB) recommended as the gold standard when a suspicious nodule is identified. Nevertheless, cytologically indeterminate results are reported in approximately 20–30% of FNAB specimens (3), carrying a malignancy risk of 5–30% according to the Bethesda classification, and may ultimately require diagnostic thyroidectomy to establish a definitive diagnosis (4).

Accurate assessment of regional lymph node status is also important, as nodal involvement influences both therapeutic strategy and patient prognosis (5). However, preoperative ultrasound imaging of central lymph nodes demonstrates limited sensitivity, with occult metastasis rates reported in up to 40.8% of clinically node-negative (cN0) patients (6). Due to the risk of significant postoperative morbidity, prophylactic central neck dissection is generally discouraged in differentiated thyroid cancer in the absence of clinical evidence of nodal involvement (7). Consequently, there remains an unmet need for reliable, non-invasive preoperative tools capable of predicting lymph node metastasis (LNM) status, with the aim of minimizing unnecessary surgical interventions (8).

Inflammation is closely associated with carcinogenesis, promoting tumor initiation, progression, and metastasis (9). Peripheral blood inflammatory indices derived from routine complete blood counts, such as the neutrophil-to-lymphocyte ratio (NLR), platelet-to-lymphocyte ratio (PLR), lymphocyte-to-monocyte ratio (LMR), and systemic immune-inflammation index (SII), have been investigated as accessible and cost-effective biomarkers for the diagnosis and prognosis of various malignancies, including thyroid cancer (10, 11). Among these, the SII has been proposed as a potentially more comprehensive indicator, as its integration of three hematological parameters enables a more thorough assessment of the balance between pro-tumorigenic inflammation and anti-tumor immunity (12).

Given the challenges associated with the preoperative diagnosis of thyroid cancer and the assessment of lymph node involvement, as well as the established link between malignancy and systemic inflammation, the SII may represent a complementary diagnostic tool.

While individual studies have investigated SII in thyroid cancer, no meta-analysis has simultaneously evaluated its diagnostic accuracy and its association with LNM within a unified analytical framework. Therefore, we conducted a meta-analysis to evaluate the clinical significance of SII in the diagnosis of thyroid cancer and in the prediction of LNM.

## 2 Methods

### 2.1 Study eligibility and data collection

A systematic literature search was performed from inception to February 20^th^, 2026, to identify all relevant sources featuring clinical data on SII in thyroid cancer. The PubMed database, Web of Science, and Scopus were explored using the following keywords: (“Systemic Immune-Inflammation Index” OR “SII”) AND (“thyroid cancer” OR “thyroid neoplasm*” OR “thyroid carcinoma” OR “thyroid nodule”). No language or publication date restrictions were applied. The reference lists of all eligible articles and relevant review articles were manually screened to identify additional studies not captured by electronic search. This systematic review was registered in PROSPERO (PROSPERO 2025 CRD420251233710).

Studies were considered eligible for inclusion if they met the following criteria: (1) enrolled patients with histopathologically confirmed thyroid cancer; (2) reported preoperative SII values calculated as (platelet count × neutrophil count)/lymphocyte count; and (3) provided sufficient data for at least one of the following: diagnostic accuracy (true positives, false positives, true negatives, and false negatives, or sensitivity and specificity with sample sizes), or mean SII values with standard deviations stratified by LNM status. Studies were excluded if they were conference abstracts without full-text availability, reviews or meta-analyses, case reports, studies on populations not representative of general thyroid cancer patients (e.g., restricted exclusively to cN0T1–T2 papillary thyroid carcinoma or exclusively to patients with Hashimoto’s thyroiditis), or if they provided insufficient data for quantitative synthesis.

Study selection was performed using the Rayyan web-based platform for systematic reviews (13). Two reviewers (R.S.D. and A.T.-S.) independently screened titles and abstracts for relevance, followed by full-text assessment of potentially eligible articles against the predefined inclusion and exclusion criteria. Disagreements were resolved by consultation with a third reviewer (B.-S.U.). Data extraction was performed independently using a standardized data collection form. The following variables were extracted from each eligible study: first author, year of publication, country of origin, study design, sample size, patient demographics (age, sex distribution), thyroid cancer histological subtype, SII cut-off values (where applicable), diagnostic accuracy data (true positives, false positives, true negatives, false negatives), mean SII values and standard deviations for lymph node metastasis and non-metastasis groups, and any reported hazard ratios or odds ratios with corresponding 95% confidence intervals. When studies reported median and interquartile range instead of mean and standard deviation, appropriate statistical methods were applied to estimate these parameters (14, 15).

### 2.2 Outcome measures

The primary outcomes of this meta-analysis were defined along two complementary clinical axes. The first primary outcome was the diagnostic accuracy of SII for distinguishing thyroid cancer from benign thyroid conditions, assessed through pooled sensitivity, specificity, positive and negative likelihood ratios (LR+ and LR−), diagnostic odds ratio (DOR), and the area under the summary receiver operating characteristic curve (AUC). These measures were derived from the 2×2 contingency tables (true positives, false positives, true negatives, and false negatives) extracted from each eligible study. The second primary outcome was the association between preoperative SII levels and LNM in thyroid cancer patients, quantified as the mean difference (MD) in SII values between patients with confirmed LNM and those without nodal involvement, with corresponding 95% confidence intervals.

Secondary outcomes included the assessment of between-study heterogeneity for both diagnostic accuracy estimates and mean difference analyses, the evaluation of publication bias through Deeks’ funnel plot asymmetry test for the diagnostic meta-analysis, and the robustness of pooled estimates as determined by leave-one-out sensitivity analyses and by excluding outlier studies identified *a priori* based on divergent effect direction or extreme data dispersion.

Risk of Bias Assessment: The methodological quality of included studies was assessed using the Newcastle-Ottawa Scale (NOS) for observational studies. Studies were evaluated across three domains: selection, comparability, and outcome/exposure. A maximum score of 9 stars was possible; studies scoring ≥7 was considered high quality.

### 2.3 Statistical analysis

All statistical analyses were performed using R software (version 4.5.1., released 13 June 2025; R Foundation for Statistical Computing, Vienna, Austria). For diagnostic meta-analysis, the bivariate Reitsma random-effects model was employed to jointly estimate pooled sensitivity and specificity while accounting for their inherent negative correlation, using the *mada* package. This model uses restricted maximum likelihood (REML) estimation and operates on the logit-transformed sensitivity and false positive rate (FPR). Summary estimates were back-transformed to the original scale for interpretation. The summary receiver operating characteristic (SROC) curve was constructed from the bivariate model, and the area under the curve (AUC) was calculated to quantify overall discriminatory ability. Pooled positive and negative likelihood ratios (LR+, LR−) and the DOR were derived from the pooled sensitivity and FPR. Between-study heterogeneity in the diagnostic meta-analysis was assessed through the variance–covariance matrix of the random effects and the correlation between logit-transformed sensitivity and logit-FPR, which quantifies the threshold effect. Supplementary univariate random-effects meta-analyses of sensitivity and specificity were conducted using the inverse variance method with logit transformation via the *meta* package, providing I² statistics and Cochran’s Q test for each measure. A leave-one-out sensitivity analysis was performed by iteratively excluding each study and re-fitting the bivariate model to evaluate the stability of the pooled estimates. Publication bias was assessed using Deeks’ funnel plot asymmetry test, which regresses the log diagnostic odds ratio against the inverse of the square root of the effective sample size. For the meta-analysis of the association between SII and LNM, the MD with 95% confidence intervals (CI) was calculated using the inverse variance method under a random-effects model (DerSimonian–Laird estimator) to account for anticipated between-study heterogeneity. Heterogeneity was quantified using the I² statistic, Cochran’s Q test (Chi²), and the between-study variance (τ²). An I² value of 0–25% was considered low, 25–50% moderate, 50–75% substantial, and >75% considerable heterogeneity. A sensitivity analysis excluding the outlier study (Refaat 2025) (19) was conducted to assess the robustness of the pooled estimate and to evaluate its impact on heterogeneity. Forest plots were generated for all analyses. A two-sided p-value < 0.05 was considered statistically significant.

## 3 Results

### 3.1 Study characteristics

**Figure 1:**
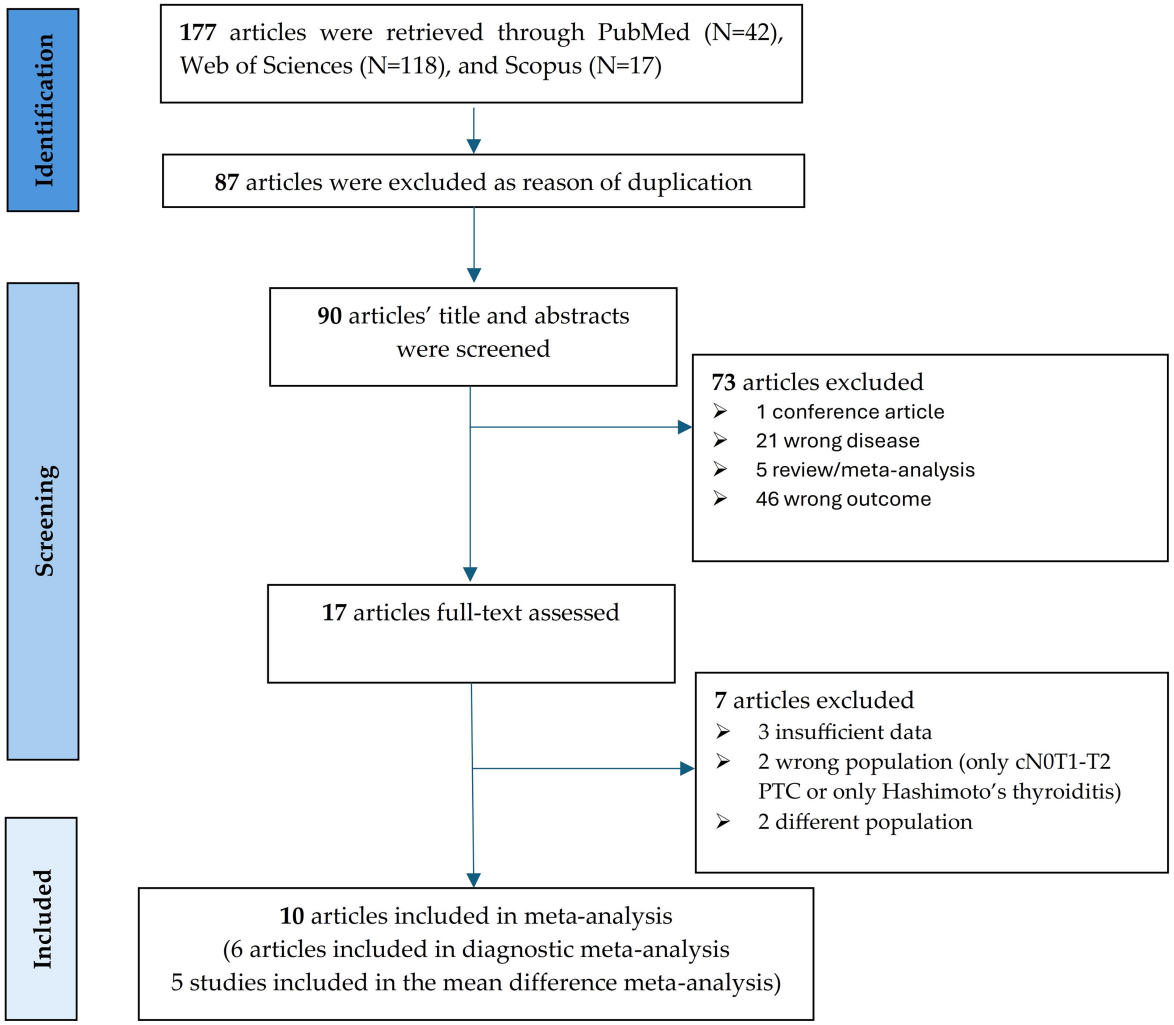
PRISMA flow.

The systematic literature search identified a total of 177 potentially relevant articles across three electronic databases: Web of Science (n = 118), Scopus (n = 17), and PubMed (n = 42). After removing 87 duplicate records, 90 unique articles remained for title and abstract screening. Of these, 73 articles were excluded for the following reasons: wrong outcome (n = 46), wrong disease (n = 21), review or meta-analysis (n = 5), and conference article (n = 1). The remaining 17 articles underwent full-text assessment for eligibility. At this stage, six additional articles were excluded due to insufficient data (n = 3), wrong population - specifically studies restricted to cN0T1–T2 papillary thyroid carcinoma or limited to patients with Hashimoto’s thyroiditis (n = 2) - and a different study population (n = 2). Ultimately, 10 articles met all inclusion criteria and were included in the quantitative meta-analysis (**Figure 1**). Of these, six studies provided sufficient data for the diagnostic accuracy meta-analysis and five studies reported mean SII values stratified by LNM status, allowing inclusion in the mean difference meta-analysis. The study selection process is summarized in the PRISMA flow diagram (**Figure 1**) and the characteristics of the studies are presented in **Table 1**.

**Table 1:** Main characteristics of individual studies included in the meta-analysis.

Study	Country	Sample size	Malignant vs. benign/LN+ vs LN-	Sex female/male	Age median/mean (range) malignant vs. benign/LN+ vs LN- groups	Study design	Cut-off (10^9/L)	Method for cut-off	NOS	Cancer type	LLNM/CLNM
Deng 2022 (16)	China	514	140/ 374	412/ 102	45/50	Retrospective	545.63	ROC curve	8	NR	NR
Gu 2024 (17)	China	547	303/ 244	465/ 82	NR	Retrospective	625.375	ROC curve	9	PTC	NR
Ozdemir 2024 (18)	Turkey	272	51/ 221	192/ 80	43.9 ± 17.3/ 48.8 ± 11.4	Retrospective	475.4	ROC curve	8	PTC	CLNM
Refaat 2025 (19)	Egypt	225	57/ 93	NR	45.5 ± 14.4/ 45.6 ± 14.2	Retrospective	366.5	ROC curve	8	Various histotypes (PTC, FTC, MTC, other)	NR
Sorrenti 2025 (20)	Italy	197	157/ 40	139/ 58	54/50	Retrospective	465.71	ROC curve	9	Various histotypes	NR
Şenoymak 2024 (21)	Turkey	607	573/ 34	484/ 123	51.3 ± 16.3/ 54 (45-62)	Retrospective	489.86	ROC curve	7	DTC	NR
Tang 2023 (22)	China	500	343/ 157	383/ 117	46/53	Retrospective	439	Median	9	PTC	NR
Vural 2023 (23)	Turkey	242	73/ 168	195/ 47	56.1 ± 11.1/ 56.8 ± 12.9	Retrospective	654.13	ROC curve	6	PTC	NR
Yang 2025 (24)	China	231	106/ 125	153/78	43.7 ± 12.9 (17-80)	Retrospective	NR	NR	9	DTC	NR
Zhao 2022 A (25)	China	702	106/ 596	529/ 173	42.3 ± 14.4/47.2 ± 12.3	Retrospective	NR	NR	9	PTC	LLNM
Zhao 2022 B (25)	China	171	22/ 149	125/ 46	41.7 ± 14.2/46.1 ± 12.5	External validation cohort	NR	NR	9	PTC	LLNM

NR, Not reported; DTC, Differentiated thyroid carcinoma; PTC, Papillary thyroid carcinoma; FTC, Follicular thyroid carcinoma; MTC, Medullary thyroid carcinoma; LLNM, Lateral lymph node metastasis; CLNM, Central lymph node metastasis.

### 3.2 Diagnostic meta-analysis

#### 3.2.1 Study characteristics and individual diagnostic accuracy

**Figure 2:**
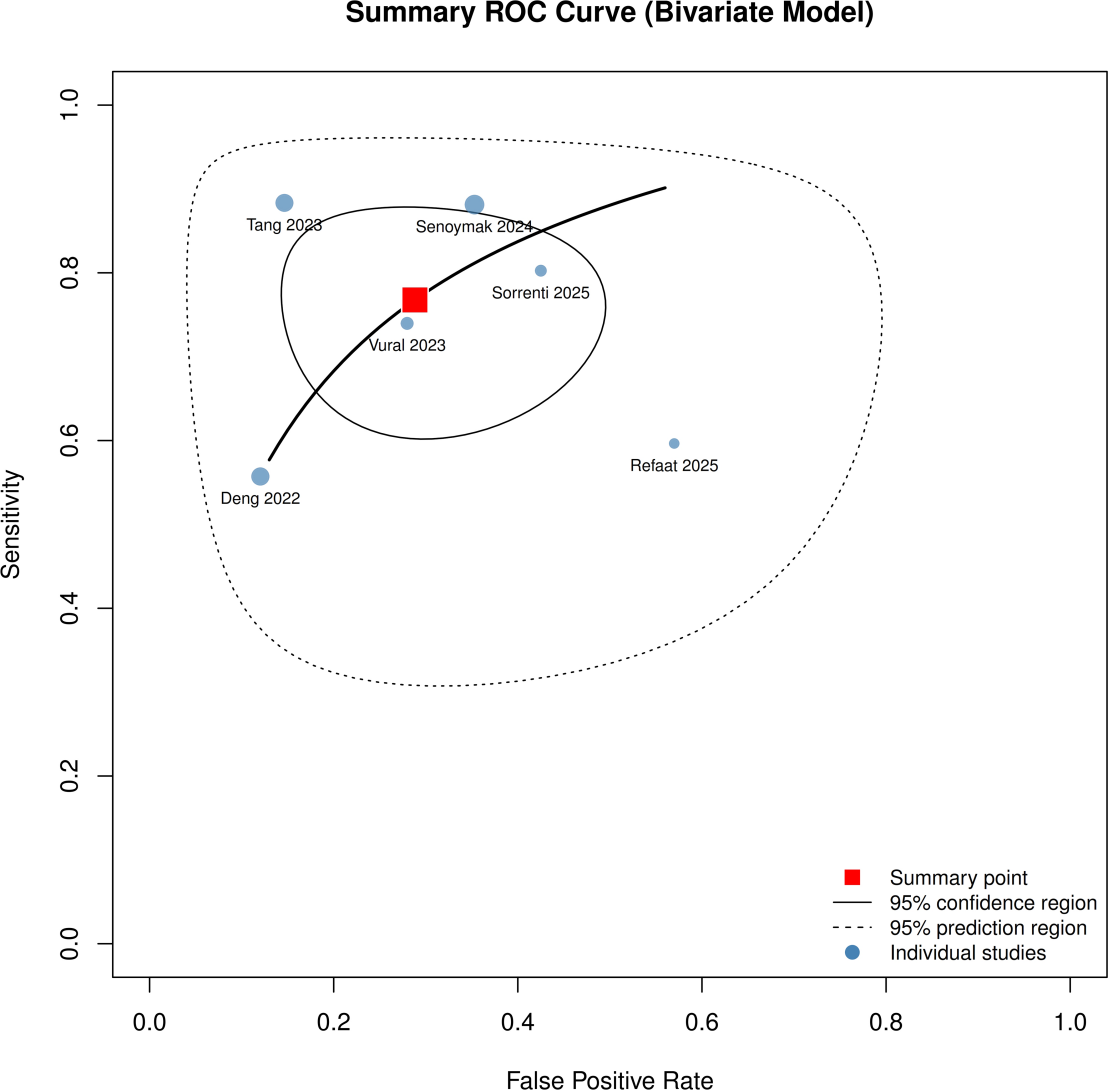
Summary receiver operating characteristic (SROC) curve for the diagnostic accuracy of the systemic immune-inflammation index (SII) in distinguishing thyroid cancer from benign thyroid conditions. Each blue circle represents an individual study, plotted by its observed false positive rate (1 − specificity, x-axis) and sensitivity (y-axis); circle area is proportional to study weight in the bivariate model. The solid black line is the SROC curve fitted by restricted maximum likelihood estimation. The red square denotes the summary operating point (pooled sensitivity = 0.768, pooled false positive rate = 0.288). The inner solid ellipse delineates the 95% confidence region around the summary point, reflecting uncertainty in the pooled estimate; the outer dashed ellipse delineates the 95% prediction region, which indicates the range within which the true sensitivity–specificity pair of a future study drawn from the same population would be expected to fall. The width of the prediction region relative to the confidence region reflects the substantial between-study variability captured by the random-effects model. The summary area under the curve was 0.805 (partial AUC restricted to observed false positive rates = 0.776).

A total of six studies (2209 patients) met the inclusion criteria for this diagnostic meta-analysis. The individual study estimates of diagnostic accuracy showed considerable variability across studies. Sensitivity ranged from 0.557 (Deng 2022) (16) to 0.883 (Tang 2023) (22), while specificity ranged from 0.430 (Refaat 2025) (19) to 0.880 (Deng 2022) (16). The DOR varied widely from 1.12 (Refaat 2025) (19) to 44.13 (Tang 2023) (22), reflecting substantial heterogeneity in diagnostic performance across the included studies, as shown in **Figure 2**.

#### 3.2.2 Pooled diagnostic accuracy (bivariate model)

**Figure 3:**
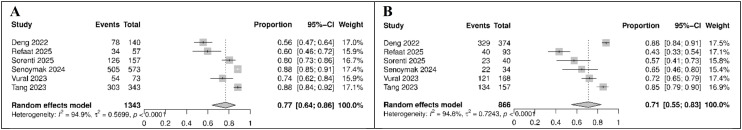
A forest plots of pooled sensitivity **(A)** and specificity **(B)** of the SII for the diagnosis of thyroid cancer. For each study, the grey square represents the point estimate (proportion), the horizontal line shows the corresponding 95% confidence interval (CI), and square area is proportional to study weight.

The bivariate (Reitsma) random-effects model was employed to jointly estimate the pooled sensitivity and specificity while accounting for their inherent correlation. The model was fitted using REML estimation. The pooled sensitivity was 0.768 (95% CI: 0.638–0.861) and the pooled specificity was 0.712 (95% CI: 0.549–0.834). The summary area under the ROC curve (AUC) was 0.805 (partial AUC restricted to observed FPRs: 0.776), indicating good overall discriminatory ability, as shown in **Figure 3**.

The positive likelihood ratio (LR+) was 2.78, suggesting that a positive test result is approximately 2.8 times more likely in patients with the condition than in those without it. The negative likelihood ratio (LR−) was 0.34, indicating that a negative test result reduces the probability of disease by approximately threefold. The pooled DOR was 8.16, confirming moderate overall diagnostic accuracy.

The hierarchical summary ROC (HSROC) model yielded consistent results, with a positive LR of 2.78, a negative LR of 0.34, and a DOR of 9.23, further corroborating the findings from the bivariate model.

#### 3.2.3 Assessment of heterogeneity

Substantial heterogeneity was observed across the included studies. In the univariate analyses, the I² statistic for sensitivity was 94.9% (τ² = 0.570; Cochran’s Q = 98.80, df = 5, p < 0.0001), and for specificity it was 94.6% (τ² = 0.724; Q = 92.35, df = 5, p < 0.0001). These values indicate very high between-study variability that cannot be attributed to chance alone.

In the bivariate model, the between-study variance was 0.571 on the logit sensitivity scale and 0.724 on the logit false positive rate scale. The correlation between logit-transformed sensitivity and logit-FPR was −0.058, suggesting only a negligible threshold effect across the included studies. The I² estimate using the Zhou and Dendukuri approach (26) was 71.8%, while the Holling sample size-unadjusted approach (27) yielded I² values of 92.8–94.1%.

#### 3.2.4 Sensitivity analysis

A leave-one-out sensitivity analysis was performed to evaluate the robustness of the pooled estimates. The results demonstrated that no single study disproportionately influenced the overall findings (**Table 2**). Pooled sensitivity ranged from 0.735 (excluding Tang 2023) to 0.804 (excluding Deng 2022), and pooled specificity ranged from 0.672 (excluding Deng 2022 or Tang 2023) to 0.759 (excluding Refaat 2025). The direction and magnitude of the pooled estimates remained consistent regardless of which study was excluded, supporting the robustness of the overall conclusions.

**Table 2:** Newcastle-Ottawa quality assessments scale.

Study	Selection				Comparability	Outcome/exposure			Score
	1	2	3	4	5	6	7	8	
Deng 2022 (16)	★	★	★		★★	★	★	★	8
Gu 2024 (17)	★	★	★	★	★★	★	★	★	9
Ozdemir 2024 (18)	★	★	★	★	★	★	★	★	8
Refaat 2025 (19)	★	★	★	★	★★	★	★		8
Sorrenti 2025 (20)	★	★	★	★	★★	★	★	★	9
Şenoymak 2024 (21)	★	★	★		★	★	★	★	7
Tang 2023 (22)	★	★	★	★	★★	★	★	★	9
Vural 2023 (23)	★	★	★			★	★	★	6
Yang 2025 (24)	★	★	★	★	★★	★	★	★	9
Zhao 2022 (25)	★	★	★	★	★★	★	★	★	9

There are the stars from the NOS score.

The sensitivity analysis excluding Refaat 2025 from the diagnostic meta-analysis yielded a pooled sensitivity of 0.76 (95% CI: 0.66-0.88) and a pooled specificity of 0.76 (95% CI: 0.63–0.86), corresponding to an LR+ of 3.39, an LR− of 0.28, and a DOR of 13.06, AUC of 0.842. Compared with the primary analysis (sensitivity 0.77, specificity 0.71, LR + 2.67, LR− 0.33, DOR 8.16), exclusion of Refaat 2025 produced modest improvements in pooled estimates and a substantial increase in the DOR, while preserving the overall direction and clinical interpretation of the findings. The robustness of the pooled estimates is therefore confirmed, and the diagnostic performance of SII does not appear to be driven by any single study.

The sensitivity analysis excluding Vural 2023, due to the NOS score of 6, from the diagnostic meta-analysis yielded a pooled sensitivity of 0.77 (95% CI: 0.61-0.88) and a pooled specificity of 0.71 (95% CI: 0.50–0.85), corresponding to an LR+ of 2.83, an LR− of 0.34, and a DOR of 9.93, AUC of 0.763. Compared with the primary analysis (sensitivity 0.77, specificity 0.71, LR + 2.67, LR− 0.33, DOR 8.16), exclusion of Vural 2023 produced modest improvements in pooled estimates and the DOR, while preserving the overall direction and clinical interpretation of the findings. The robustness of the pooled estimates is therefore confirmed, and the diagnostic performance of SII does not appear to be driven by any single study.

#### 3.2.5 Publication bias

**Figure 4:**
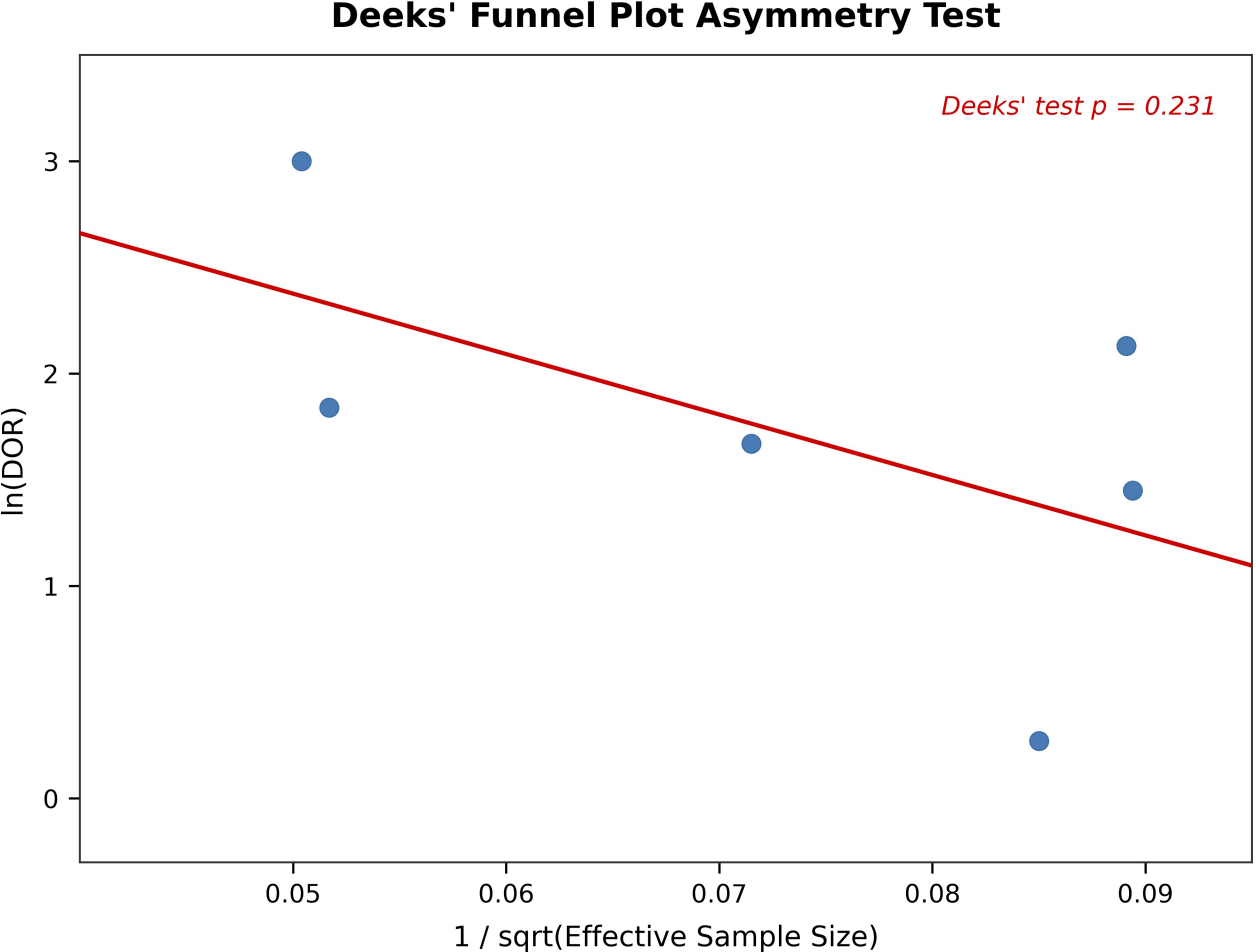
Deeks’ funnel plot asymmetry test for the assessment of publication bias in the diagnostic meta-analysis of the SII in thyroid cancer. The diagnostic log odds ratio (ln DOR, y-axis) is plotted against the inverse of the square root of the effective sample size (1/√ESS, x-axis); each blue point represents one study. The red line is the linear regression of ln DOR on 1/√ESS. Symmetry of the plot and a non-significant slope are interpreted as evidence against publication bias.

Deeks’ funnel plot asymmetry test was performed to assess potential publication bias. The regression of log DOR against the inverse of the square root of the effective sample size showed no statistically significant asymmetry (p = 0.231), suggesting that the likelihood of publication bias is low, as shown in **Figure 4**.

### 3.3 Association between SII and LNM

#### 3.3.1 Characteristics of included studies

Five studies involving a total of 2,073 patients were included in this meta-analysis evaluating the association between the SII and LNM in thyroid cancer. Among the total study population, 645 patients had confirmed LNM and 1,428 patients had no evidence of LNM. The individual study sample sizes ranged from 73 (Zhao 2022b) (25) to 547 (Gu 2024) (17) participants in the LNM and noLNM groups combined.

#### 3.3.2 Pooled mean difference in SII between LNM and noLNM groups

**Figure 5:**
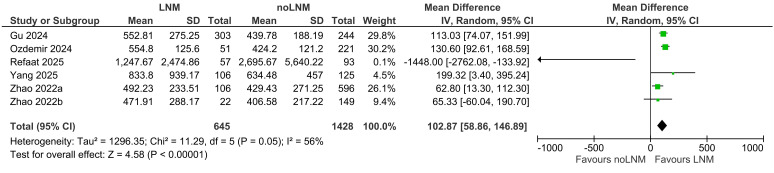
Forest plot of the association between the Systemic Immune-Inflammation Index and lymph node metastasis in thyroid cancer. For each cohort, the green square represents the MD (mean SII in the LNM group minus mean SII in the noLNM group), the horizontal line denotes its 95% confidence interval (CI), and square area is proportional to study weight (calculated as the inverse of within-study variance). MD, mean difference; CI, confidence interval; LNM, lymph node metastasis. Random-effects model (inverse variance method) was used.

The pooled analysis demonstrated that SII levels were significantly higher in thyroid cancer patients with LNM compared to those without, as shown in **Figure 5**. The overall mean difference (MD) was 102.87 (95% CI: 58.86–146.89; Z = 4.58; p < 0.00001), indicating that patients with LNM had, on average, SII values approximately 103 units higher than patients without LNM. The forest plot revealed that the pooled effect estimates clearly favored higher SII values in the LNM group, with the confidence interval excluding zero.

#### 3.3.3 Individual study findings

Five of the six included studies reported higher SII values in the LNM group compared to the noLNM group. The two most heavily weighted studies, Ozdemir 2024 (18) (weight: 30.2%) and Gu 2024 (17) (weight: 29.8%), both demonstrated statistically significant positive associations with mean differences of 130.60 (95% CI: 92.61–168.59) and 113.03 (95% CI: 74.07–151.99), respectively. Zhao 2022a (25) (weight: 26.1%) also showed a significant positive association (MD = 62.80; 95% CI: 13.30–112.30), while Yang 2025 (24) reported a larger but less precise effect (MD = 199.32; 95% CI: 3.40–395.24). Zhao 2022b (25) yielded a non-significant result (MD = 65.33; 95% CI: −60.04–190.70), likely due to its small LNM sample (n = 22).

Notably, Refaat 2025 (19) was a clear outlier, reporting a mean difference of −1,448.00 (95% CI: −2,762.08 to −133.92), indicating that SII was substantially lower in the LNM group in this study. This result was in the opposite direction to all other studies and carried a negligible weight in the pooled analysis (0.1%), owing to its extremely large standard deviations (SD = 2,474.86 in the LNM group and 5,640.22 in the noLNM group), suggesting marked data dispersion in this study.

#### 3.3.4 Heterogeneity assessment and sensitivity analysis

Moderate heterogeneity was observed across the included studies. The Cochran’s Q test yielded a Chi² of 11.29 (df = 5, p = 0.05), and the I² statistic was 56%, indicating that more than half of the total variability in effect estimates was attributable to between-study heterogeneity rather than sampling error. The between-study variance (τ²) was estimated at 1,296.35.

**Figure 6:**
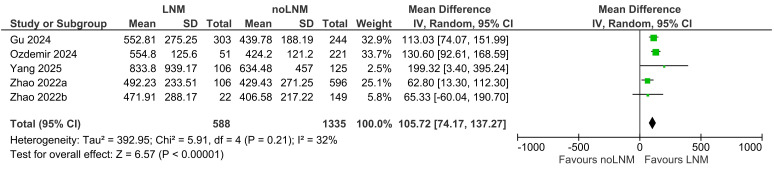
Forest plot of the association between the systemic immune-inflammation index and lymph node metastasis in thyroid cancer in sensitivity analysis, excluding Refaat 2025.

As Refaat 2025 (19) exhibited markedly divergent results from the remaining studies - reporting a paradoxically lower SII in the LNM group (MD = −1,448.00) with exceptionally large standard deviations (SD = 2,474.86 and 5,640.22 for the LNM and noLNM groups, respectively) - a sensitivity analysis was performed by excluding this study, as shown in **Figure 6**. The pooled analysis of the remaining five studies (588 LNM vs. 1,335 noLNM; total n = 1,923) yielded a mean difference of 105.72 (95% CI: 74.17–137.27; Z = 6.57; p < 0.00001), which was comparable in magnitude to the original pooled estimate (MD = 102.87) but with a notably narrower confidence interval and stronger statistical significance. Importantly, the exclusion of Refaat 2025 (19) substantially reduced heterogeneity, with the I² decreasing from 56% (Chi² = 11.29, df = 5, p = 0.05) to 32% (Chi² = 5.91, df = 4, p = 0.21), and the between-study variance (τ²) dropping from 1,296.35 to 392.95. This indicates that the moderate heterogeneity observed in the primary analysis was largely attributable to this single outlier study. The consistency of the pooled mean difference before and after exclusion (102.87 vs. 105.72), coupled with the substantial improvement in homogeneity, confirms that the overall finding of significantly elevated SII in thyroid cancer patients with LNM is robust and not driven by any individual study.

#### 3.3.5 PTC-restricted sensitivity analysis

**Figure 7:**
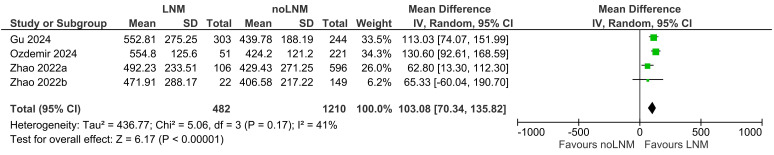
Forest plot of the association between the systemic immune-inflammation index and lymph node metastasis in thyroid cancer in papillary thyroid carcinoma-restricted sensitivity analysis.

Because papillary thyroid carcinoma represents both the histological subtype of greatest epidemiological relevance and the predominant histology across the included studies, a pre-specified sensitivity analysis was performed restricting the meta-analysis to studies that enrolled exclusively, or near-exclusively, PTC patients. Three studies contributing four cohorts met this criterion (Gu 2024, Ozdemir 2024, Zhao 2022a, and Zhao 2022b; 482 LNM vs. 1,210 noLNM patients; total n = 1,692). The pooled mean difference was 103.08 (95% CI: 70.34–135.82; Z = 6.17; p < 0.00001), virtually identical to the primary analysis (MD = 102.87) and to the analysis excluding Refaat 2025 (MD = 105.72). Between-study heterogeneity was reduced compared with the primary analysis (τ² = 436.77; Cochran’s Q Chi² = 5.06, df = 3, p = 0.17; I² = 41%, down from 56%), and the confidence interval was narrower than in the primary analysis (width 65.5 vs. 88.0). All PTC studies demonstrated positive mean differences favoring higher SII in the LNM group, with statistical significance reached in three of the four cohorts (Gu 2024, Ozdemir 2024, Zhao 2022a). The remaining cohort (Zhao 2022b) showed a positive but non-significant effect (MD = 65.33; 95% CI: −60.04 to 190.70), consistent with its small LNM subgroup (n = 22) and correspondingly limited statistical power (**Figure 7**).

## 4 Discussion

This study presents a meta-analytic evaluation of the SII in thyroid cancer, addressing two complementary clinical questions: the diagnostic accuracy of SII for distinguishing thyroid cancer from benign thyroid conditions, and its association with LNM as a marker of disease aggressiveness, based on evidence from ten studies totaling 4,282 participants.

In the diagnostic setting, the pooled sensitivity of 76.8% and specificity of 71.2% indicate that SII alone is not sufficiently accurate to serve as a standalone diagnostic test for thyroid cancer. Approximately one in four patients with malignancy would be missed (false negative), and nearly three in ten patients without cancer would be incorrectly flagged (false positive). However, the negative likelihood ratio of 0.34 suggests that SII has particular value in reducing post-test probability when the result is negative. In clinical practice, a low SII value in a patient with an indeterminate thyroid nodule combined with low-suspicion ultrasonographic features, could strengthen the clinician’s confidence in recommending surveillance over immediate surgery, thereby reducing unnecessary invasive procedures.

The pooled diagnostic estimates derived from this meta-analysis should be regarded as preliminary and interpreted with considerable caution, in light of three convergent considerations. First, between-study heterogeneity was very high (I² > 94% for both sensitivity and specificity in the univariate analyses, and I² = 71.8% in the bivariate model using the Zhou and Dendukuri approach (26)), exceeding the conventional threshold for considerable heterogeneity. Under these conditions, the pooled estimates describe an average effect across markedly heterogeneous populations rather than a stable, transportable measure of diagnostic accuracy, and their direct extrapolation to any individual clinical setting is not warranted. Second, the SII cut-off values used to define a positive test varied nearly two-fold across studies (366.5–654.13 × 10^9^/L), reflecting the absence of a standardized threshold. This threshold variability likely accounts for a substantial proportion of the observed between-study variability, since higher cut-offs mechanically increase specificity at the expense of sensitivity. Third, the reference standard was not uniformly applied: while malignant cases were confirmed by histopathology across all studies, benignity of control nodules was established histopathologically in only a subset, with the remainder relying on Bethesda II cytology and/or imaging follow-up. This partial verification bias systematically inflates pooled specificity, because cytologically benign nodules harboring occult malignancy are misclassified as true negatives; the same caveat propagates to the derived measures (LR+ and DOR). Pooled specificity should therefore be interpreted as an upper bound rather than as an unbiased estimate of true diagnostic performance. Taken together, these considerations support framing SII as a hypothesis-generating biomarker requiring prospective validation in studies with standardized cut-offs and uniform histopathological reference standards, rather than as a diagnostic tool ready for incorporation into current clinical decision algorithms.

In the preoperative risk stratification, the significantly elevated SII in patients with LNM (MD = 102.87) supports its potential incorporation into preoperative risk stratification models. These findings are exploratory and hypothesis-generating and should not be interpreted as a basis for clinical decision-making at this stage. Nonetheless, if confirmed in prospective studies, elevated preoperative SII might serve as an adjunctive signal prompting more comprehensive preoperative imaging evaluation in patients with thyroid cancer. Whether SII could eventually inform surgical planning, including the extent of lymph node evaluation, remains to be established through prospective, adequately powered studies with standardized protocols. Conversely, patients with low preoperative SII and favorable clinicopathological features may be candidates for a more conservative approach, thereby reducing surgical morbidity including recurrent laryngeal nerve injury and hypoparathyroidism (7).

Taken together, these findings indicate that SII may possess both diagnostic and predictive relevance in thyroid cancer: it can moderately discriminate between malignant and benign thyroid pathology, and among patients with confirmed malignancy, higher preoperative SII values are associated with a greater likelihood of LNM.

These findings are consistent with the association between thyroid cancer and systemic inflammation, highlighting SII as a reflection of the underlying immunological imbalance that characterizes malignant thyroid disease (28). Neutrophilia promotes tumor progression through increased production of reactive oxygen species, exerting protumorigenic effects via DNA mutagenesis and suppression of antitumor immunity. Additionally, neutrophil-derived MMP-9 induces secondary VEGF release from the extracellular matrix, facilitating tumor invasion and metastatic spread (29). Thrombocytosis facilitates metastasis through platelet aggregation around circulating tumor cells, shielding them from immune surveillance (30). Lymphocytopenia reflects suppression of cytotoxic immunosurveillance, reducing the host’s capacity to eliminate malignant cells (31). Collectively, the convergence of these hematological alterations, as captured by the SII, underscores the systemic immunological dysregulation that accompanies thyroid malignancies and supports metastatic dissemination.

A notable finding of this study is the divergent heterogeneity profiles between the two meta-analyses. The diagnostic meta-analysis exhibited very high heterogeneity (I² > 94% for both sensitivity and specificity in univariate analyses; I² = 71.8% by the Zhou and Dendukuri bivariate approach), whereas the LNM association meta-analysis demonstrated moderate heterogeneity (I² = 56%). This discrepancy is informative and requires careful interpretation.

The high heterogeneity in the diagnostic meta-analysis likely reflects several converging factors: threshold variability across studies (SII cut-offs ranging from 366.5 to 654.13), differences in nodule size as a possible confounding variable given its potential correlation with inflammatory markers (32), and inconsistent control group composition — with most studies using benign nodules, but one incorporating multinodular goiter and autoimmune thyroiditis patients (23). The nearly two-fold difference in reported cut-off values (366.5–654.13) likely accounts for a substantial proportion of the observed between-study variability in sensitivity and specificity, as higher thresholds increase specificity at the expense of sensitivity. Reference standard inconsistency further introduced verification bias, as surgical histopathology was not uniformly applied to benign group across all six diagnostic studies. Given this heterogeneity, SII should be regarded as a complementary tool alongside ultrasound and FNAB, rather than a standalone diagnostic marker.

Exclusion of the study Refaat 2025 from the diagnostic synthesis improved the pooled estimates (sensitivity from 0.77 to 0.79; specificity from 0.71 to 0.76) and increased the diagnostic odds ratio from 8.16 to 13.06, suggesting that the primary pooled estimates may represent a conservative lower bound on the true diagnostic performance of SII. Importantly, the direction of effect and the clinical interpretation of the findings remained unchanged, supporting the robustness of the primary analysis. The convergence of evidence across both meta-analyses identifying Refaat 2025 as an outlier — and the substantial improvement in pooled estimates following its symmetric exclusion — addresses the methodological concern about asymmetric outlier handling and reinforces confidence in the meta-analytic conclusions.

In the LNM meta-analysis, the moderate I² of 56% is largely driven by the outlier study of Refaat 2025 (19), which reported a paradoxically large negative association with implausibly high standard deviations, and was consequently assigned negligible weight (0.1%) by the random-effects model. Excluding this study, the remaining studies showed consistently elevated SII in the LNM group. An additional source of heterogeneity was the inconsistent reporting of LNM location, as most studies did not distinguish between CLNM and LLNM, precluding subgroup analysis despite the distinct prognostic implications of these two compartments (33).

The PTC-restricted sensitivity analysis directly addresses concerns that histological heterogeneity might have driven the primary findings. Restricting the synthesis to studies enrolling exclusively or near-exclusively PTC patients yielded a pooled mean difference virtually indistinguishable from the primary estimate (103.08 vs. 102.87), with reduced between-study heterogeneity (I² 41% vs. 56%) and a narrower confidence interval. This convergence has two implications. First, it indicates that the association between elevated SII and LNM observed in the primary analysis is not an artefact of mixing histological subtypes but is robustly present within the PTC population - which is reassuring given that PTC accounts for over 85% of thyroid malignancies and is the subtype for which preoperative risk stratification has the greatest clinical impact. Second, the modest reduction in heterogeneity (from substantial to moderate) suggests that histological mix contributes only marginally to between-study variability in this meta-analysis, with other factors - such as differences in the definition of LNM (central vs. lateral compartment), surgical extent (therapeutic vs. prophylactic lymph node dissection), and patient selection (consecutive vs. selected cohorts) - likely accounting for the residual variability. The persistence of a clinically meaningful effect size (≈103 units, well above the within-study SDs typically reported for SII in benign thyroid disease) within a histologically homogeneous PTC population strengthens the biological plausibility of SII as a marker reflective of the inflammatory milieu that accompanies nodal spread in differentiated thyroid carcinoma.

Furthermore, variability in histological subtypes across studies, with most including predominantly papillary thyroid carcinoma but some incorporating other subtypes, may have contributed to heterogeneity, given that the relationship between SII profiles and histological subtype of thyroid malignancy remains incompletely characterized (34). Although individual studies generally reported excluding patients with hematological or inflammatory comorbidities, the absence of uniform exclusion criteria across studies cannot be entirely discounted as a source of variability.

Several other systemic inflammatory indices have been investigated in thyroid cancer, including the NLR and PLR. The NLR captures only the neutrophil–lymphocyte axis, and the PLR captures only the platelet–lymphocyte axis, whereas SII synthesizes both dimensions into a single index, offering a theoretically more comprehensive reflection of the systemic immune-inflammatory state. For illustrative purposes only, a recent meta-analysis of NLR and PLR in thyroid cancer reported pooled sensitivity and specificity of 75% and 62% for NLR, and 70% and 57% for PLR, which appear broadly comparable to, or marginally lower than, the corresponding estimates for SII in the present analysis (sensitivity of 76.8% and specificity of 71.2%) (35). This juxtaposition must, however, be interpreted with substantial caution: the NLR/PLR estimates derive from a separate meta-analysis built on a different study pool, with non-overlapping included cohorts, distinct cut-off values, and differing reference-standard definitions. Such an indirect comparison is therefore descriptive rather than statistical and provides no formal evidence that any one index outperforms the others. Determining whether SII offers genuine added value over NLR and PLR will require direct head-to-head comparisons performed within identical patient cohorts, using a common reference standard and pre-specified cut-off values — ideally as a pre-planned secondary objective in prospective diagnostic accuracy studies.

Our findings should be interpreted in the context of the recently published meta-analysis by Liu and Xiao (2026) (36), which evaluated the role of nutritional (PNI, CONUT) and SII in predicting LNM and prognosis of thyroid cancer. Their pooled analysis of 13 studies reported that elevated SII was only marginally associated with increased risk of LNM using hazard ratios (HR: 1.00; 95% CI: 1.00–1.01; I² = 93%), leading the authors to question its clinical significance. Our meta-analysis, by contrast, adopted a fundamentally different analytical approach - pooling mean differences in absolute SII values between LNM and noLNM groups rather than hazard ratios — and demonstrated a robust and clinically meaningful association (MD = 105.72; 95% CI: 74.17–137.27; p < 0.00001; I² = 32%). Several factors may explain this apparent discrepancy. First, the HR-based approach employed by Liu and Xiao treats SII as a continuous variable per unit increase, which inherently yields effect estimates close to unity (HR ≈ 1.00 per one-unit increment) when the index spans a wide numerical range, as is the case with SII values typically ranging from 200 to over 2,000. This does not imply a negligible association but rather reflects the scale of measurement. Second, the very high heterogeneity in their analysis (I² = 93%) compared to ours (I² = 32%) suggests that the HR-based approach may be more susceptible to variability introduced by differing cut-off values, adjustment covariates, and modeling strategies across studies. Third, our study complements Liu and Xiao’s work by adding a diagnostic accuracy dimension that was not addressed in their meta-analysis, demonstrating that SII also has moderate discriminatory ability for distinguishing malignant from benign thyroid conditions (AUC = 0.805). Taken together, the two meta-analyses are not contradictory but rather complementary: while the per-unit hazard ratio suggests a modest incremental risk, the mean difference analysis reveals that patients with LNM carry a substantially and significantly higher SII burden — on average approximately 106 units greater — than those without nodal involvement. This convergence of evidence from different analytical perspectives strengthens the overall conclusion that SII is meaningfully associated with LNM in thyroid cancer.

Beyond the diagnostic and predictive roles evaluated in the present study, Chen et al., 2025 demonstrated that elevated SII is an independent predictor of postoperative recurrence in follicular thyroid carcinoma, suggesting that its clinical relevance may extend beyond the preoperative setting (37). Longitudinal studies examining the dynamic changes in SII following treatment, including surgery, radioactive iodine therapy, and thyroid hormone suppression, could further establish its value as a monitoring biomarker for disease recurrence.

This study has some limitations. First, both meta-analyses included a small number of studies, which limits the precision of pooled estimates and heterogeneity quantification, and precludes meaningful subgroup analyses or meta-regression to formally explore sources of variability. Second, the majority of included studies were retrospective in design, with only one incorporating a prospective validation cohort, and residual confounding by unmeasured variables, such as histological subtype, disease stage, or laboratory methodology cannot be excluded. Third, the absence of individual patient data prevents identification of optimal SII cut-off values for either diagnostic or prognostic applications and precludes receiver operating characteristic analysis to determine clinically actionable thresholds. The nearly two-fold variation in reported cut-off values across diagnostic studies (366.5–654.13) underscores this limitation and likely contributed to the very high heterogeneity observed in the diagnostic meta-analysis (I² > 94%). Fourth, the included studies originated exclusively from China, Turkey, Italy, and Egypt, which may limit the generalizability of the findings to other populations with different ethnic backgrounds, dietary patterns, and baseline inflammatory profiles.

A further limitation of the diagnostic meta-analysis concerns differential verification bias resulting from non-uniform reference standard application across the six included studies. While Sorrenti 2025, Tang 2023, and Vural 2023 confirmed both malignant and benign groups by surgical histopathology, Şenoymak 2024 and Refaat 2025 used FNAB (Bethesda II) as the only reference standard for the benign group, and Deng 2022 did not clearly specify the proportion of patients confirmed by each method, reporting only that patients underwent surgical treatment or FNAB without specifying proportions. Given that the risk of malignancy for Bethesda II cytology is estimated at 0–3%, a small proportion of cytologically benign nodules in the affected studies may have harbored occult malignancy, potentially contaminating the benign group and introducing uncertainty in diagnostic accuracy estimates. However, this design reflects the current standard of care: the 2023 European Thyroid Association Clinical Practice Guidelines for thyroid nodule management recommend surveillance without surgical confirmation for Bethesda II nodules, reserving surgery for Bethesda V–VI cytology or symptomatic disease (38). Performing thyroidectomy for research verification in patients with benign cytology would therefore be ethically unjustifiable.

Finally, it should be noted that SII values may be influenced by a range of non-oncological factors, including acute infections, autoimmune disorders, chronic inflammatory conditions, obesity Other influences could bepharmacological treatments such as corticosteroids or immunosuppressants, which were not uniformly controlled for across the included studies and may have introduced additional unmeasured confounding. Despite these limitations, to our knowledge, this is the first meta-analysis to comprehensively evaluate SII in thyroid cancer from both a diagnostic and a predictive perspective within a unified framework.

Future research should prioritize prospective, multi-center designs with standardized SII protocols, histological subtype-specific analyses, separate reporting of CLNM and LLNM, and direct head-to-head comparisons with other inflammatory indices to establish the incremental value of SII in thyroid cancer management. Also, future prospective, multi-center validation studies should report SII separately by histological subtype (PTC vs. FTC vs. MTC) and by LNM compartment (CLNM vs. LLNM), which the current literature does not permit.

## 5 Conclusions

This comprehensive meta-analysis highlights that SII holds clinical value in thyroid cancer both as a diagnostic marker and as a predictor of LNM. The convergence of these findings suggests that SII captures clinically meaningful aspects of the systemic inflammatory response relevant to both tumor presence and tumor aggressiveness. However, substantial heterogeneity and the limited number of included studies temper these conclusions. SII should be regarded as a promising, inexpensive complementary biomarker within a multi-parameter risk assessment framework, rather than a replacement for established diagnostic modalities.

## Data Availability

The original contributions presented in the study are included in the article/supplementary material. Further inquiries can be directed to the corresponding author.
